# Complications after superficial parotidectomy for pleomorphic adenoma

**DOI:** 10.4317/medoral.22386

**Published:** 2018-06-21

**Authors:** Pedro Infante-Cossio, Eduardo Gonzalez-Cardero, Alberto Garcia-Perla-Garcia, Enrique Montes-Latorre, Jose-Luis Gutierrez-Perez, Eugenia Prats-Golczer

**Affiliations:** 1 Professor, Department of Surgery, School of Medicine, University of Seville, Department of Oral and Maxillofacial Surgery, Virgen del Rocio University Hospital, Seville, Spain; 2 Staff Surgeon, Department of Oral and Maxillofacial Surgery, Virgen del Rocio University Hospital, Seville, Spain; 3 Staff, Department of Clinical Neurophysiology, Virgen del Rocio University Hospital, Seville, Spain; 4 Professor, Department of Stomatology, School of Dentistry, University of Seville, Department of Oral and Maxillofacial Surgery, Virgen del Rocio University Hospital, Seville, Spain; 5 Staff Surgeon, Department of Oral and Maxillofacial Surgery, Torrejon University Hospital, Madrid, Spain

## Abstract

**Background:**

The significance of complications after superficial parotidectomy remains unclear, since prospective studies are lacking. The aim of this study was to evaluate facial nerve dysfunction and other postoperative complications after superficial parotidectomy for pleomorphic adenoma of the superficial lobe and to identify the associated risk factors.

**Material and Methods:**

Prospective and descriptive clinical study on 79 patients undergoing formal superficial parotidectomy with the modified facelift incision, dissection of the facial nerve and reconstruction with the superficial musculoaponeurotic system flap. Function of the facial nerve using the House-Brackmann scale and the intra- and postoperative complications were recorded at 1 week and 1, 3, 6 and 12 months. A descriptive, inferential and binary logistic regression analysis were performed for the variables facial nerve dysfunction, tumor size and location, clinical presentation and duration of surgery.

**Results:**

77.2% of the patients presented facial paresis at 1 week, with the marginal-mandibular branch being the most commonly affected (64.5%). 94.9% recovered the facial function at 6 months and 100% at 12 months. A statistically significant relationship was found between the appearance of facial paresis and tumor location in the superior lateral area of the superficial lobe, size >2 cm and prolonged operative time. None of the remaining variables showed significant differences at any study timepoint. At 12 months, 57% of patients had recovered tactile sensitivity in the earlobe. The clinical occurrence of Frey’s syndrome was 11.4%.

**Conclusions:**

Despite the high incidence of postoperative facial paresis at 1 week, its magnitude was low and the recovery time was short. Tumor location in the parotid superficial lobe upper area, size and prolonged operative time are risk factors that can worsen facial paresis at different study timepoints. The knowledge of these complications is relevant for patient´s counseling and to achieve better long-term outcomes.

** Key words:**Superficial parotidectomy, pleomorphic adenoma, parotid gland, facial nerve paralysis, postoperative complications.

## Introduction

Conventional treatment for pleomorphic adenoma located in the superficial lobe of the parotid gland consists of a formal superficial parotidectomy to remove the tumor with a clear margin preserving the facial nerve. The elimination of the superficial lobe ensures a very low incidence of tumor recurrence, but it can cause a number of major and minor complications due to the dissection of the facial nerve and the loss of at least three quarters of the parotid gland volume (1). The most significant complication is the facial nerve dysfunction that can appear as paralysis (complete loss of function) or paresis (partial loss) (2,3). In most cases, this facial paresis is temporary and recovery of nerve function is achieved before 6 months after surgery, usually within the first two months (4,5). Other postparotidectomy complications include infection, salivary fistula, hemorrhage, hematoma, sialocele/seroma, aesthetic deformity, numbness around the earlobe and Frey’s syndrome (2,6-8).

Contributing factors associated with complications after superficial parotidectomy remain unclear. The incidence varies considerably from one study to another, and in general, most of them are retrospective and lack standardized methods to assess postoperative complications. In recent years, some centers have advocated more conservative alternative procedures such as partial parotidectomy and extracapsular dissection, in order to limit or avoid dissection of the facial nerve and preserve part of the superficial lobe of the parotid gland (9,10). Other surgeons have used the facelift incision (rhytidectomy), first described for a parotidectomy by Appiani, using a retroauricular incision and positioning the cut close to the hairline to disguise the most visible part of the scar and thus improve the aesthetic outcomes (11). Several methods, such as reconstruction with the superficial musculoaponeurotic system (SMAS) flap, have been used to cover the postoperative defect after parotidectomy (8). In addition, the SMAS flap appears to prevent the occurrence of Frey’s syndrome (12).

The knowledge of the potential risks and complications associated with superficial parotidectomy may be relevant to offer better preoperative counseling to patients, improve preoperative planning and achieve better long-term results. The objective of this prospective study was to evaluate the facial nerve dysfunction and other surgical complications related to the formal superficial parotidectomy for pleomorphic adenoma located in the superficial lobe of the parotid gland, and to identify the possible associated risk factors.

## Material and Methods

A descriptive and prospective longitudinal clinical study was conducted in the Department of Oral and Maxillofacial Surgery of the Virgen del Rocio University Hospital, Seville (Spain), on patients undergoing superficial parotidectomy with dissection of the facial nerve for pleomorphic adenoma located in the superficial area of the parotid gland. We excluded patients aged <18 years, tumors with another type of histology or with extension to the deep lobe, and patients with previous operation on the parotid gland. The study was approved by the hospital ethics committee and all patients gave their informed consent to participate.

Preoperatively, the diagnosis of pleomorphic adenoma was established in all cases by a combination of fine needle aspiration cytology and computed tomography or magnetic resonance imaging. All surgical procedures met two conditions: removal of the superficial lobe and dissection of the facial nerve. A modified facelift incision was designed and a SMAS flap was raised. Next, the main trunk of the facial nerve was identified at its exit from the stylomastoid foramen and all its peripheral branches were followed in an antegrade manner, while the superficial lobe was excised. When technically possible, the posterior branch of the great auricular nerve (GAN) was preserved. After the parotidectomy had been completed, the SMAS was re-approximated and sutured back to the anterior border of the sternocleidomastoid muscle, a suction drain was placed, and the skin incision was closed.

**Figure 1:**
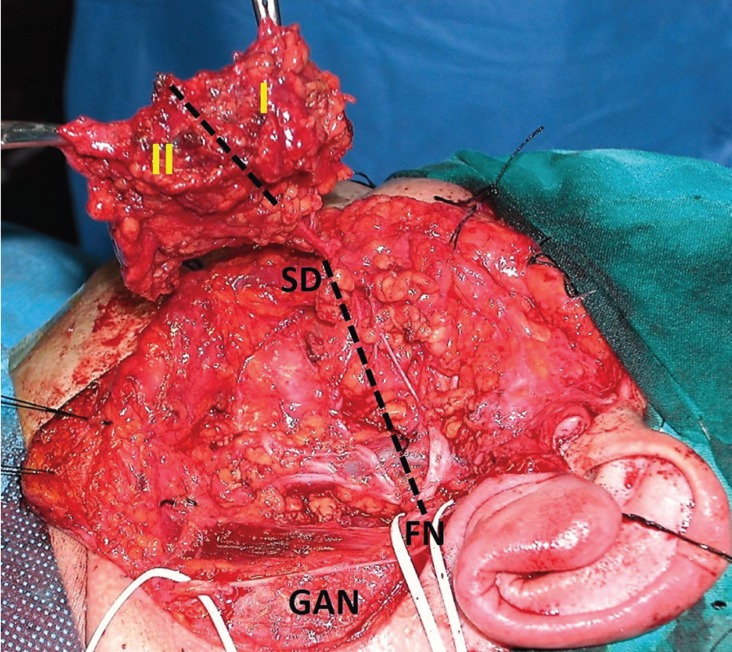
Intraoperative view of areas I and II after dissection of the facial nerve (FN). Detail of the facial nerve and GAN dissection, while the superficial lobe remains attached by the Stensen duct (SD).

- Data collection. The following pre- and intraoperative demographic and clinical data were included: age and sex of patients, size and location of the tumor, mode of clinical presentation, duration of the operation, affected facial side, and preservation of the GAN. The location of the tumor was evaluated by dividing the superficial lobe into two anatomical areas: I (superior lateral) and II (inferior lateral) (10). Tumors located above a line drawn from the bifurcation of the facial nerve to the exit of the Stensen duct were assigned to area I, and those below this line, to area II (Fig. 1). Basically, area I corresponded to the temporo-zygomatic branches and area II to the marginal-mandibular-cervical branches.

Postoperative complications such as facial paralysis, local complications (wound infection, salivary fistula, hemorrhage, hematoma, sialocele/seroma), numbness around the earlobe, aesthetic deformity, and Frey’s syndrome, were prospectively recorded by one of the authors in follow-up visits at 1 week and 1, 3, 6 and 12 months. The function of the facial nerve was graded with the House-Brackmann facial nerve grading system (HBFNGS). The HBFNGS is a widely validated instrument (13,14) that classifies the grade of clinical paresis from grade I (no paresis) to grade VI (total paresis). For this study, it was considered that an affectation of grade II or above in either of the nerve branches had a clinical paresis of the facial nerve. An electromyography was performed to compare the non-paretic side with the paretic side of the face. For the sensory alteration of the GAN, the tactile sensitivity was evaluated in the earlobe and pre-auricular skin, and it was graduated in 3 grades (no loss of sensitivity, hypoaesthesia, dysesthesia). Cutaneous scarring was classified into 3 categories (ideal, moderate, hypertrophic). The satisfaction of the patient with the cosmetic outcome was evaluated by means of an ordinal visual analog scale (VAS) (0=intolerable, 1-3=deficient, 4-6=average, 7-9=good, 10=normal or very good). The deformity caused by the depression of the facial contour was classified in 3 grades (none, moderate, major).

- Statistical analysis.

The collected data were analyzed in SPSS for Windows v.19 (SPSS Inc. USA) by an independent medical statistician. For descriptive statistics of qualitative variables, absolute and relative frequencies were used and, for quantitative variables, the median or interquartile range if they did not follow a normal distribution as assessed by the Kolmogorov-Smirnov test. For the qualitative variables, a bivariate study was performed using the Chi-Square test or the Fisher´s exact test, as appropriate. The comparison of the quantitative variables was assessed using the Student’s t-test for independent samples and the non-parametric Mann-Whitney U-test if they followed an asymmetric distribution. Values of *p*<0.05 were considered significant.

Results

## Results

We included 79 patients in the study, 39 men (49.4%) and 40 women (50.6%), with a mean age of 48 years (range 24-81 years). The mean time of follow-up was 30.65 months (range 12-45 months).  Table 1 shows the preoperative demographic and clinical data. 65.8% % of tumors were located on the right side while 35.5% were sited in area I, 53.1% in area II, and 11.4% involved both superficial areas (I and II). The most common clinical presentation was as a slowly growing mass (84.9%). The mean tumor size was 2.44 cm (range 1-6 cm). The mean time until the patient’s first visit was 18 months (range 2-180 months). The mean operative time was 154.5 minutes (range 85-240 minutes, SD=57.8). The median time of hospitalization was 2.5 days (range 1-7 days).

**Table 1:**
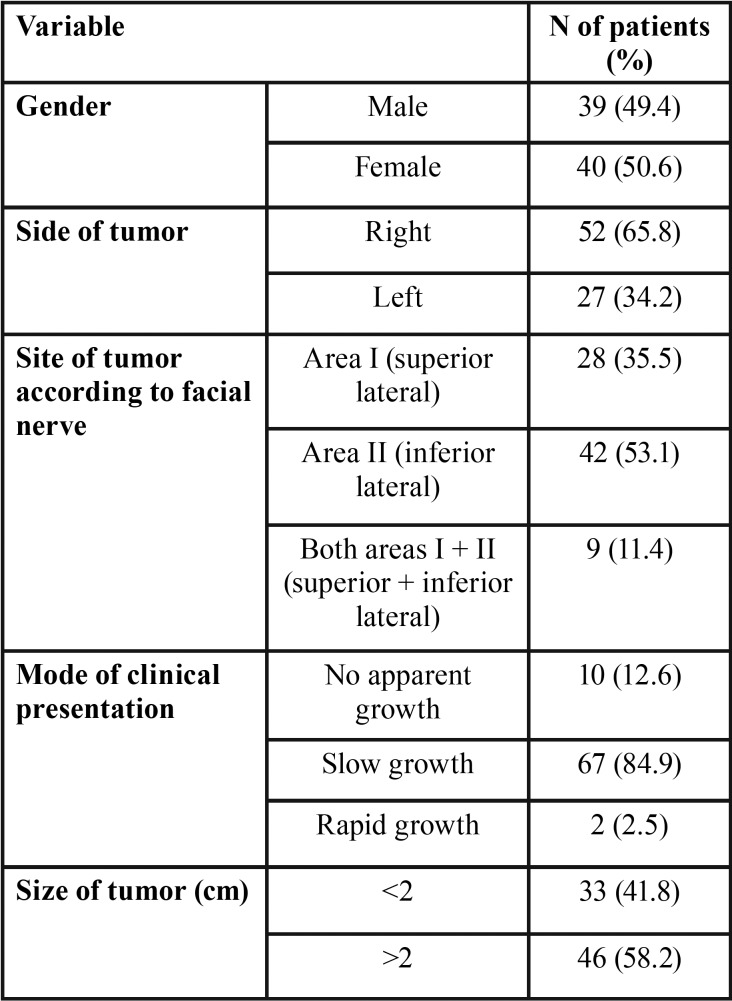
Preoperative demographic and clinical characteristics of 79 patients.

According to the HBFNGS, 77.2% of the patients showed a grade II of involvement or above in either of the facial nerve branches at 1 week, 51.9% at 1 month, 30.4% at 3 months, 5% at 6 months and 0% at 12 months ( Table 2). The mean recovery time was 2 months (interquartile range=0.25-5.75). The paresis for each branch at each timepoint of the study revealed that the marginal-mandibular one was the most frequently affected at 1 week (64.5%) and had the highest percentage of affectation at all the timepoints. The paresis was 0% at 12 months for all branches. At 1 week, the magnitude of the paresis was graded as grade II in 80.3%, grade III in 18.1% and grade IV in 1.6%. In multivariate analysis, paresis of the facial nerve showed a statistically significant difference with the location of tumor in area I at 1 week (*p*=0.002) and 1 month (*p*=0.004), size of tumor >2cm at 6 months (*p*=0.040), and prolonged operative time >154.5 minutes at 1 week (*p*=0.016), 1 month (*p*=0.002) and 3 months (*p*=0.018). Facial paresis showed no statistical relationship at any timepoint of the study with respect to age, sex, mode of clinical presentation and affected side.

**Table 2:**
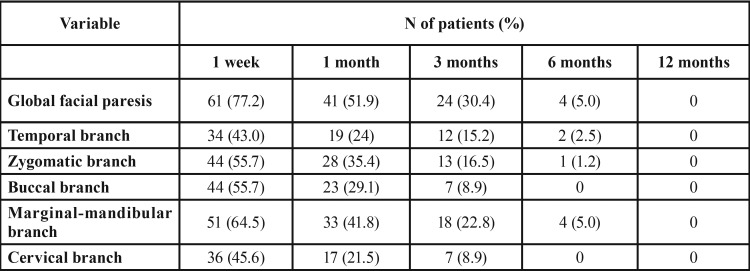
Paresis of the facial nerve and of each branch after superficial parotidectomy according to the HBFNGS, at each timepoint of the study.

The neurophysiological study revealed a lower amplitude on the side of the dissected nerve in 98.3% of cases at 1 week (48.15% of amplitude, SD=25.9%). The cases without clinical paresis had a 61% of amplitude compared to the non-dissected side, and the cases with clinical paresis had a 43.5% of amplitude compared to the non-dissected side. The difference in amplitude between both sides was statistically significant (*p*=0.017). The neurophysiological determination revealed a complete recovery of all patients at 12 months.

**Figure 2:**
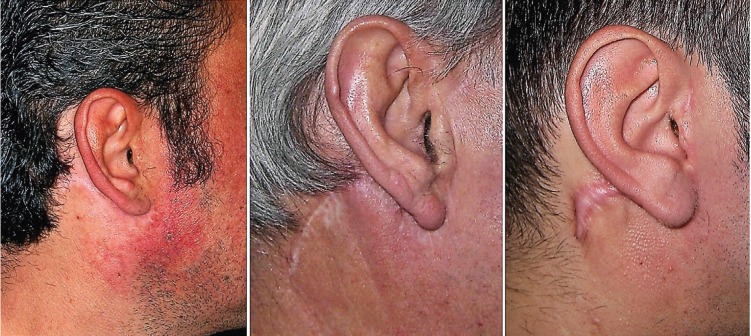
Scar appearance at 1 year. Ideal (left), moderate (central) or hypertrophic (right).

**Figure 3:**
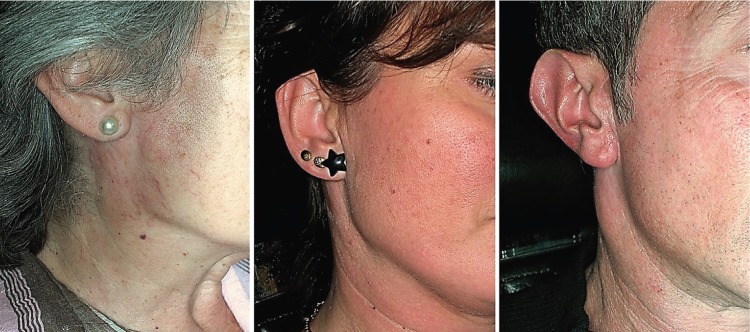
Depression of the facial contour at 1 year. None (left), moderate (central) or major (right).

 Table 3 shows the incidence of other complications. 12.7% of patients presented hematoma at 1 week. The posterior branch of the GAN was preserved in 41 patients (51.9%). 67 patients (84.9%) exhibited a reduction in tactile sensitivity in the earlobe or preauricular skin at 1 week. At 12 months, 45 patients (57%) recovered the same grade of sensitivity as before surgery, and the rest had some grade of numbness. 61% of the patients in whom the GAN had been preserved and 45.6% in whom it had not, recovered the sensitivity, although the differences were not statistically significant. The recovery time for those patients who recovered the sensitivity of the GAN at 12 months was 7.03 months (SD=4.15 months). At 12 months, 75.9% of patients had an ideal scar (Fig. 2). The mean patient´s satisfaction score with the scar was 9 in the VAS (interquartile range=9-10). 43% of the patients did not notice any deformity caused by the depression of the facial contour and 40.5% referred a moderate depression (Fig. 3). 9 patients (11.4%) exhibited Frey’s syndrome, although only 1 patient reported symptoms without being asked specifically.

**Table 3:**
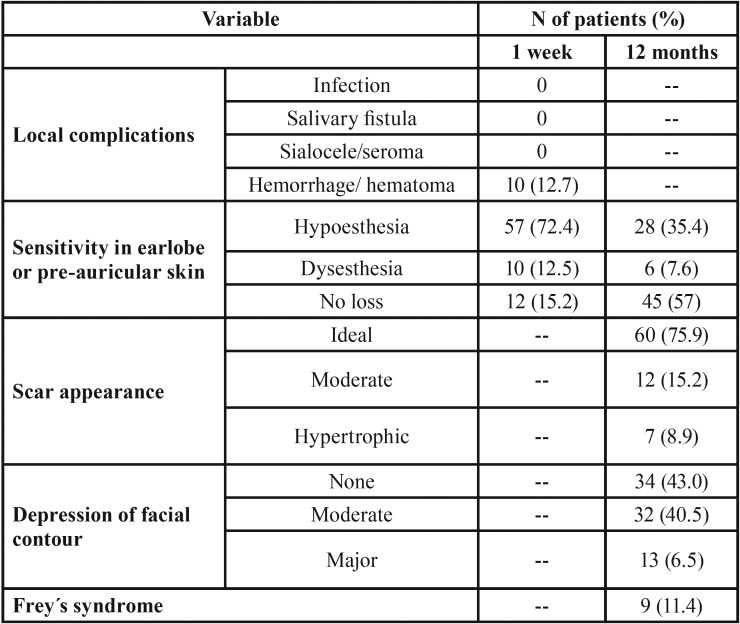
Complications after superficial parotidectomy (excluding facial nerve paresis).

## Discussion

Pleomorphic adenoma is the most common benign neoplasm of the parotid gland (15). It occurs most frequently between the ages of 40 and 50 years, and in women than in men (16). It usually appears as a slowly growing painless mass, with a variable time evolution, normally located in the caudal portion of the superficial lobe. In general terms, the demographic and clinical findings of our study confirm all these data, widely previously reported in the literature.

Our findings showed that facial nerve dysfunction was the most common complication after superficial parotidectomy for pleomorphic adenoma. Although the percentage of appearance of facial nerve paresis at 1 week was 77.2%, half of the patients recovered facial function at 1 month, 94.9% at 6 months and 100% at 12 months, by and large demonstrating a short recovery time. In the literature, the percentage of facial postoperative paresis varies from 10% to 70% for transient involvement, and from 0 to 19% for definitive involvement (1,2,17-19).

The high incidence of postoperative facial paresis in our study was possibly due to the fact that we considered as paresis that minimal involvement of II grade or above in either of the facial nerve branches according to the HBFNGS. Given that we recorded the data in a prospective manner and by specific branch, our results are difficult to compare because most of studies are retrospective and measured the global percentage of facial paresis without considering the magnitude of paresis or the specific branch involved. The most affected branch at 1 week was the marginal-mandibular one (64.5%), as in most of the series (1,2).

Despite the high incidence of postoperative facial paresis of patients at 1 week, its magnitude was low (80.3% had grade II according to the HBFNGS). For statistical analysis, the evaluation of the grade of paresis was not taken into account because our objective was to evaluate the appearance of clinical paresis and its evolution in the timepoints of the study, regardless of the grade and the affected branch. Although the majority of tumors were located in the inferior area of the superficial lobe (53.1%), we did not find a statistical relationship between dysfunction of the marginal-mandibular branch and location in the inferior lateral site. Our results suggest that the location of tumor in the superior area of the superficial lobe, the size of tumor >2cm and the prolonged operative time are risk factors that can worsen facial paresis at different study timepoints.

According to previous studies, several factors have been associated with a higher incidence of facial paresis in parotid surgery (2,17,20-25): extent and type of surgical procedure (higher incidence in total parotidectomy and greater resection of glandular tissue), patient age, operative time, tumor histopathology (higher incidence in malignant tumors), tumor size (greater incidence in large tumors) and location (higher incidence in deep lobe tumors), previous parotid surgery (higher incidence in cases of recurrent disease) and presence of intraglandular inflammation. The significance of these findings remains controversial, because some studies (including the present one) confirm some factors and others do not. On the one hand, only a few studies are prospective and comprise a significant number of patients with a standardized follow-up that allow the true assessment of facial nerve dysfunction (19). On the other hand, some studies include several tumor etiologies and types of parotid surgery. To minimize these biases, the 79 patients monitored in our study were prospectively and regularly recorded, underwent the same standardized surgical operation by the same surgeons, and were operated on for the same condition (pleomorphic adenoma).

Electromyography records the spontaneous and voluntary muscular electrical responses caused by the electrodes placed on the facial mimic muscles. It allows to assess the function of the facial nerve on the paretic side compared to the non-paretic side of the face. This procedure is easy, affordable and useful for the initial diagnosis of facial paralysis and the monitoring of long-term recovery. In 98.3% of patients, a lower amplitude of nerve function was recorded on the affected side at 1 week, which was associated with the appearance of clinical paresis. Our results demonstrated a statistical association between the amplitude of the paretic side and the recovery time of the paresis.

The GAN was always visualized during parotid surgery, and the integrity of its posterior branch was preserved when viable. The true meaning of preserving the GAN is controversial and some authors argued that its preservation during parotid surgery is unnecessary (26). The affectation of the GAN can cause hypoesthesia in the earlobe and pre-auricular skin, which interferes with daily habits such as kissing, shaving or wearing earrings or glasses, and an uncomfortable feeling when exposed to cold (27). 84.9% of the patients suffered a sensory deficit at 1 week, regardless of whether the nerve was preserved or not, a percentage similar to that of other studies that report a hypoesthesia in the affected area despite preserving the posterior branch of the GAN (28). Nor did we find statistical differences in the recovery of tactile sensitivity depending on it would have been preserved or not, as reported in the literature (28). Min *et al.* (28) reported that 57% of patients recovered the sensitivity at 1 year, which increased to 61% in patients who had the GAN preserved, figures very similar to ours, although they were not statistically significant. Our findings support that the preservation of the posterior branch of GAN whenever possible (29,30), although a postoperative recovery occurs in more than half of the cases at 1 year, regardless of preservation of the GAN or not. Possibly the collateral branches are those that compensate this deficit, as well as the auriculotemporal and occipital nerves.

The follow-up time for the evaluation of the esthetic outcome was 12 months, enough time to ensure complete maturation of the scar. The modified facelift incision allowed adequate access to the parotid gland to expose the facial nerve and identify the GAN, while achieving a better aesthetic outcome than the classic Blair incision once it was concealed under the hairline and folds of the skin and neck. The finding of 8.9% of hypertrophic scarring in our study is consistent with that reported by other authors (31). Some degree of alteration of the healing process was detected in younger patients (mean=39.4 years) compared to the elderly (mean=49.4 years), although this relationship was not statistically significant (*p*=0.081). The outcome according to the patient’s perception of the appearance of the scar indicated a very significant satisfaction in most of them (VAS=9).

The superficial lobe comprises 70% to 80% of the volume of the parotid gland (32), so that after parotidectomy there is some degree of facial contour deformity that may compromise the aesthetic outcome. Reconstruction procedures such as the sternocleidomastoid muscle flap or SMAS flap are particularly indicated in younger patients and in defects larger than 3 cm (33,34). In our study, the degree of depression of the facial contour was not related to the tumor size (*p*=0.674). Despite the controversy on the use of the SMAS flap following a parotidectomy, we indicated it to prevent facial depression and improve the contour (8). In our study, the grade of depression of the facial contour was classified as moderate or major in 47% of the patients. De Vicente *et al.* (12) investigated the modified facelift incision combined with an SMAS flap on patients undergoing superficial parotidectomy, concluding that it improved the cosmetic appearance of the scar, prevented facial depression and reduced the incidence of Frey’s syndrome.

Frey´s syndrome is a significant complication after parotid surgery, showing an incidence that varies greatly from 6% to 96% (35,36). In general, it is recognized that approximately 10% of patients complain actively of Frey’s syndrome at 6 to 12 months, a percentage that increases to 20-30% if it is specifically investigated (19,37). In our study, the diagnosis was based on the spontaneous complaint of the patient, since our objective was to analyze the complications with clinical relevance. We detected an occurrence of 11.4% of Frey’s syndrome. This low occurrence can be explained by the relatively short follow-up time of 12-month and by the use of the SMAS flap in all cases. Several authors have recommended reconstruction with a SMAS flap to reduce the incidence of Frey’s syndrome (11,34), although others have not found any differences (38). None of our patients required additional treatment, since the symptoms did not concern them in any way.

Among the main limitations of our study, we must mention the lack of a control group and the relatively small size of the sample, since it is a single-center study, so the results should be interpreted with caution. The method we used to measure facial dysfunction was the HBFNGS, which although widely used and well-documented in the literature, may present intra-observer variations since it is a subjective scale. Finally, in future studies, other procedures for treatment of pleomorphic adenoma, such as extracapsular dissection or partial parotidectomy, could be considered for comparisons.

## Conclusions

The results of our study suggest that superficial parotidectomy with the modified facelift incision and reconstruction with SMAS is a safe procedure, with few complications at the long-term. Despite the high incidence of postoperative facial paresis at 1 week, its magnitude was low and the recovery time was short. The location of pleomorphic adenoma in the upper area of the parotid superficial lobe, the size >2cm and the prolonged operative time are risk factors that can worsen facial paresis at different study timepoints. More than half of the patients recovered the sensitivity of the GAN, irrespective of whether it was preserved or not. There was a low occurrence of clinical Frey´s syndrome, which did not require treatment. The knowledge of the potential risks and complications associated with superficial parotidectomy are relevant for preoperative planning, counseling to patients and to achieve better long-term outcomes.
